# Security Measures with Enhanced Behavior Processing and Footprint Algorithm against Sybil and Bogus Attacks in Vehicular Ad Hoc Network

**DOI:** 10.3390/s21103538

**Published:** 2021-05-19

**Authors:** Krzysztof Stępień, Aneta Poniszewska-Marańda

**Affiliations:** Institute of Information Technology, Lodz University of Technology, Wolczanska 215, 90-924 Łódz, Poland; krzysztof.stepien.1@p.lodz.pl

**Keywords:** vehicular ad hoc network, security, sensors, Internet of things, bogus attack, Sybil attack, enhanced behavior processing

## Abstract

Vehicular ad hoc networks (VANETs) are created according to the principles of ad hoc mobile networks (MANETs), i.e., spontaneous creation of a wireless network for vehicle-to-vehicle (V2V) communication. Each vehicle in this network is treated as a node that is part of the mobile network. VANET turns all cooperating vehicles into a wireless router or node. This makes it possible to connect all cars within range to a stationary unit and create a wide network with a huge range. VANET is widely used for better traffic management, vehicle-to-vehicle communication, and road information provision. The VANET network is exposed to identity and information attacks, concealing or delaying data transmission, or information theft. Therefore, there are multiple types of attack, such as Sybil or bogus, that might harm the whole network infrastructure. The consequences of the mentioned two attacks could lead not only to the given infrastructure but could cause hammering people’s lives. In this paper, we analyze the ongoing methods for preserving Sybil and bogus attacks in a VANET network together with the authors’ methods: the Bogus & Sybil Trust Level & Timestamp (B&STL&T) algorithm and the Bogus & Sybil Enhanced Behavior Processing & Footprint (B&SEBP&F) algorithm. The first algorithm, the Bogus & Sybil Trust Level & Timestamp (B&STL&T) algorithm was improved into the Bogus & Sybil Enhanced Behavior Processing & Footprint (B&SEBP&F), presented in the paper. The proposed methods were tested with multiple scenarios using different variations of bogus and Sybil attack and various attacker–victim node number ratios. During analysis, it was observed that detection of all attackers in the network was reduced by approximately 30% in comparison to previous work and that of other cited authors.

## 1. Introduction

The vehicle ad hoc network (VANET) is an innovative field that can improve driving safety, traffic efficiency, and safety management by information transmission between cars and public infrastructure through different communication mechanisms, such as vehicle-to-vehicle (V2V) and vehicle-to-infrastructure (V2I) communication. Systems such as IEEE 802.11p were designed to meet the requirements of ITS architectures in order to achieve the above. Although different aspects of VANETs are being examined, there is much interest in initiating the deployment of this system.

Cars are linked together in vehicle networks through ad hoc formation to form a wireless network called ad hoc vehicle networks. Typical VANETs are composed of roadside units (RSUs), onboard units (OBUs), proxy servers, and servers for administration and computers, vehicles, registry authorities, and location-based applications. There is a traffic center made up of both private and public sector firms, and every roadside unit (also known as roadside infrastructure) is connected to the transport center.

The VANET network model is based on a two-level network model where the trusted authority (TA) is set as the first level and the RSUs and vehicles are set as the second level. It could be described as follows:

- **Trusted Authority**—TA is strongly trusted by all network parties and has appropriate computing, networking, and storage capabilities. It also registers RSUs and vehicles, generating initial security parameters for all vehicles and RSUs after a successful connection.
- **Roadside Unit**—RSUs reflect the deployment of stationary infrastructure on the road, intersections, and bus stops, serving the interface between TA and vehicles—authenticating vehicle traffic messages and forwarding them to the center of authority.
- **Vehicle**—each car unit is fitted with an onboard unit (OBU) that enables the vehicle to interact with other cars and stationary units.

**Figure 1:**
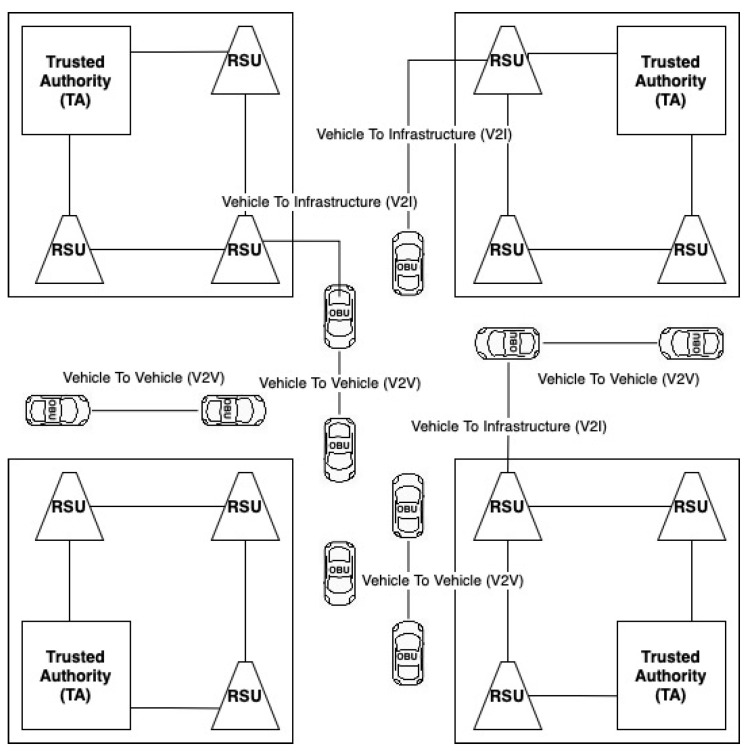
The idea of VANET network functioning.

A vehicular ad hoc network is created by applying the principles of a mobile ad hoc network (MANET)—spontaneous creation of a wireless network of mobile devices in the field of vehicles. In such networks, cars can exchange information with each other and with stationary receivers. VANET can use any type of wireless technology; however, the most commonly used is WLAN. There are many potential benefits of a VANET network (Figure 1). It can, for example:

- take action to initiate braking when the car ahead is beginning to brake,
- use platooning (a method for driving a group of vehicles together),
- provide ongoing information about traffic congestion, and
- accelerate the call to public services in the event of accidents.

The problems of such a network include the ability to manage multiple vehicles at the same time and defend against external attacks; in the event of overload, errors, or breaking into the system, a life-threatening situation for users arises.

The initial development process of any VANET network should deal with security issues. In this paper, we focus on Sybil and bogus attacks, among different security issues.

Douceur [1] first introduced the Sybil attack in the context of peer-to-peer networks. In a Sybil attack, the attacker creates a large number of false identities with which he can propagate the messages to other network participants. It poses a serious security risk as the attacker can send false messages and simulate his location (the attacker may claim to be in multiple places at once) [2]. Due to the increased number of vehicles, it is possible to carry out a DDoS attack by overloading other neighboring units. Moreover, in voting-based systems, an attacker can easily outvote the real participants, leading to a decision in his favor. The Sybil attack presents a significant threat to VANET as a successful vehicle could claim to have witnessed an accident or a traffic jam, which would alter the proposed route for any other vehicle. This could result in enormous costs for the network and disruptions to data continuity [3].

Another sort of attack that should also be taken into account is the bogus attack. Bogus information attack is a type of attack on the VANET network consisting in sending the false information through the network (hoax road event favoring traffic diversion) to gain personal benefits [4]. In this attack, the attacker can be outsider/intruder or insider/legitimate user. The attacker broadcasts false information in the vehicular network to affect the decisions of other vehicles by spreading the false information in the network. For example, a vehicle can imitate a heavy traffic on one road to prevent another vehicle from choosing that road. This attack is an example of application attack [5].

The main contribution of the paper is the introduction of new algorithm: Bogus & Sybil Enhanced Behavior Processing & Footprint (B&SEBP&F), which is the continuation of the previous one, Bogus & Sybil Trust Level & Timestamp (B&STL&T) mentioned in [6]. The new algorithm was also tested using new road events and compared with the previous one. The results of the tests were presented and analyzed in the paper.

The paper is organized as follows. In Section 2, we describe the selected aspects of bogus attack in the VANET network and the created solution to monitor the node behavior and interaction with other nodes. In Section 3, we present the selected aspects of a Sybil attack with the algorithm implemented inside the authors’ VANET network. Section 4 deals with the prototype algorithm against bogus and Sybil attacks (B&STL&T). In Section 5, we present the results obtained from new algorithm and compares it with its previous version and the solutions presented by other authors. Finally, Section 6 draws the discussion and conclusions.

## 2. Related Work on Protection against Bogus Attacks

**Figure 2:**
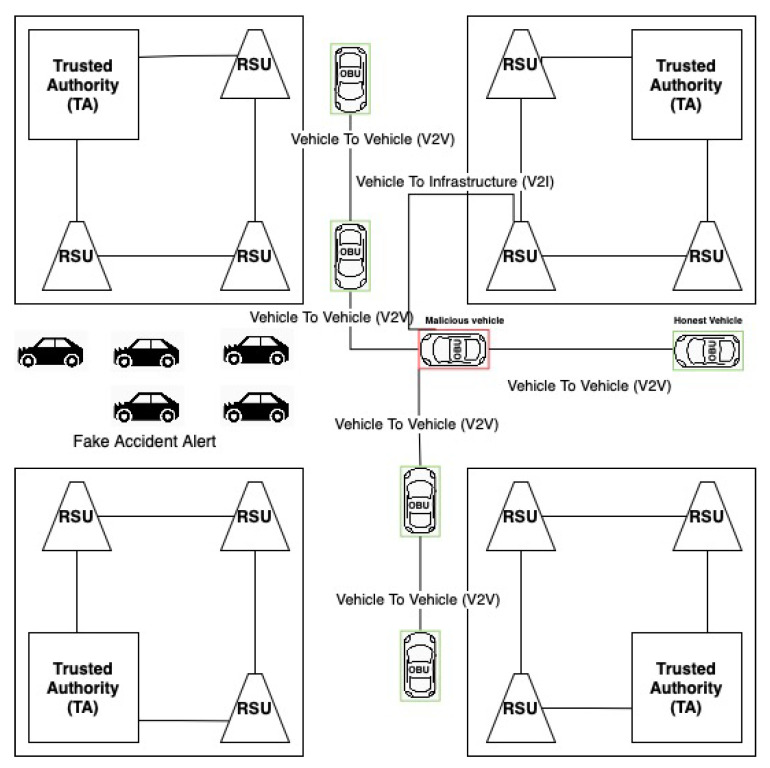
Example of bogus information attack.

One of the VANET network attacks that involves sending false information to obtain personal benefits is a bogus attack. The attacker could be an outside (i.e., intruder) or an inside (i.e., legitimate) user in this assault [7]. In order to manipulate the behavior of other vehicles, the intruder broadcasts false information in the vehicular network by disseminating false network data. For instance, to prevent any vehicle from selecting a certain route, a vehicle may mimic heavy traffic on one road (Figure 2) [8].

As an example, Figure 2 demonstrates the real congestion situation where victim cars propagate the real behavior of the current situation. It shows the false congestion alert propagated by the attacker node, although other vehicles display no congestion messages. Therefore, to identify and avoid these false messages, the particular mechanism has to be implemented.

The attacker chooses a victim node in the attack scenario and then prepares an RREQ (route request) or beacon packet for the victim’s generated AODV (ad hoc on-demand distance vector protocol) and GPSR (greedy perimeter stateless routing protocol) [9]. The attacker generates packets for a randomly selected target victim node and broadcasts these packets every five seconds on behalf of the victim car. The attacker absorbs traffic and drops any packets transmitted through him by being the newest node or the nearest node to the destination in both AODV and GPSR. This attack may also be used to isolate a node from the network, but due to the rapidly changing topology of VANETs, it will have a limited impact [10].

Several solutions provide protection against bogus attacks. The most popular are:

- Asymmetric cryptography—there is one public key in the entire network and each device has its own private key with which it signs its messages [11].
- Trust level—attempts to detect a bogus attack on the simulation network is based on assigning and modifying a particular vehicle’s level. This level is used to recognize the truthfulness of the message sent by the vehicle [12]. All vehicles are assigned to a confidence level, which is verified when determining the correctness of the obtained information [13].

Kim et al. [14] suggest a message filtering model to detect the misleading info efficiently. The model contains a threshold curve and occurrence certainty (CoE) curve. The CoE, which shows the trust level of the request data, is determined by integrating data from different sources, such as local sensors, RSUs, and reputing mechanisms [15]. A particular event program could adjust source priorities in order to mitigate computation. For instance, a road condition alert from a nearby trustworthy RSU might bypass other sources and make it impossible for them to be reviewed. The more a vehicle encounters a specific incident, the more notifications it receives to record an occurrence that raises the CoE value [16].

One of the solutions mentioned in [17] was based on the TRIP (trust-and-reputation-infrastructure-based proposal). This algorithm for traffic analysis identifies the malicious nodes in the VANET network that spread false or altered data through the network. Alert messages and regular messages are sent to another node that tests the reputation and credibility of the sender’s node [18]. The received messages are dropped if the node turns out to be malicious. Probabilistic reasoning identifies a node based on the value derived from three pieces of information [19]: past credibility rating, cars surrounding it, and the central body’s guidelines. The system proposed three values for estimating confidence values: untrusted (rejects all packet from the given node), trustworthy (accept without sending packets), and reliable (accept and send all information) [20].

One of the solutions that could prevent the sending of false information is RABTM [21] (road-site unit and beacon-based trust management) model—a framework based on BTM (beacon-based trust management) and RSU (road-site unit) [22]. It requires the use of two distinct confidence methods: indirect and direct. In order to determine the location, speed, and the direction in which the signal transmission object travels, the indirect approach uses beacon signals [23]. Trust coefficient is determined, which is then compared with the current data and then decides its credibility [24].

Another approach named the misbehavior detection system (MDS) was developed by Raya et al. [25] to identify adjacent attackers who are disseminating false details. It is believed that there is a certificate authority (CA) offering one public/private key pair and certification in each car [26]. They often use entropy, a standard measure of data, to give the opportunity for the vehicle to view and evaluate normal and irregular actions in order to identify the intruder [27].

Ghosh et al., in [28] suggested a comprehensive scheme to identify malicious vehicles for the post-crash warning application. The method used, in the first place, observes the driver’s behavior after a crash warning call [29]. The observed mobility and predicted trajectory of the vehicle with the crash mobility model is measured. If the gap between the two reaches a certain threshold value, the alarm is assumed to be incorrect [30].

Lee et al., in [31] suggested a novel credibility management system focused on misbehavior (MBRMS) that involves three components: (a) misbehavior identification, (b) rebroadcast occurrence, and (c) global eviction algorithms for the identification and filtration of incorrect facts in VANETs. Each vehicular node manages an incident information system and related activities to detect misbehavior of the node [32]. The described method used the outlier identification strategy and misbehavior of the risk value of the poor node to calculate the risk level. MBRMS easily recognizes and eliminates misbehavior nodes [33].

As it could be seen, the authors in their findings indicated multiple solutions based on the improving authentication security. They also analyzed the drivers’ behaviors on the road (Table 1).

## 3. Related Work on Protection against Sybil Attacks

**Figure 3:**
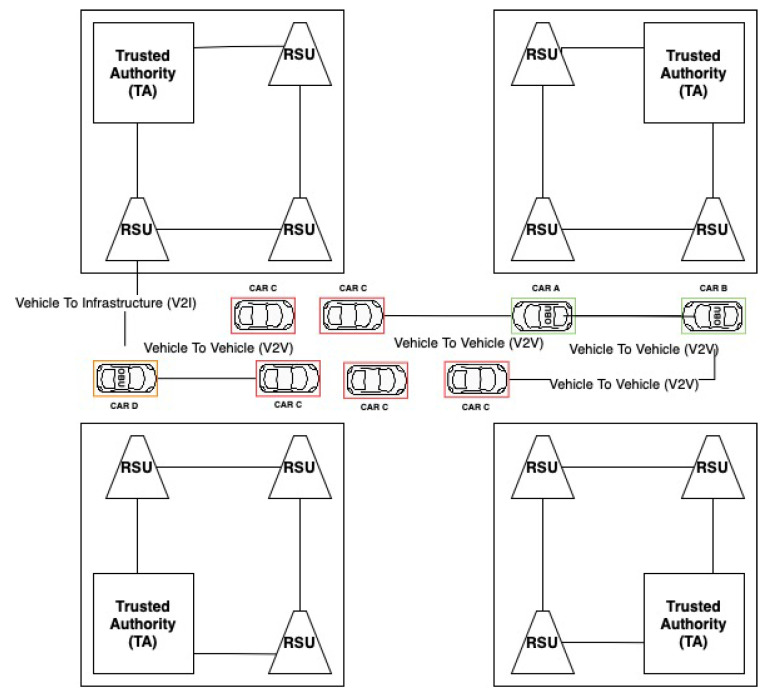
Sybil attack in VANET.

In a Sybil attack, the attacker creates a large number of false identities with which he can propagate messages to other network participants. This poses a serious security risk as the attacker can send false messages and simulate his location (the attacker may claim to be in multiple places at once) [35]. Due to the increased number of vehicles, it is possible to carry out a distributed denial-of-service (DDoS) attack by overloading other neighboring units [36]. Moreover, in voting-based systems, an attacker can easily outvote the real participants, leading to a decision in his favor. The Sybil attack presents a significant threat to VANET, as a successful vehicle could claim to have witnessed accident or traffic jam, which would alter the proposed route for any other vehicle. It could result in enormous costs for the network and disruptions to data continuity (Figure 3).

The misbehavior detection schemes (MDS) method proposed in 2011 by Sushimt Ruj in [37] was based on the detection of messages on the web and forged vehicles by examining the stream of reported information. Each entity has to decide whether the information received is true or not; the decisions are made based on consistency with other received messages. The algorithm does not use the voting system, so it is resistant to massive attacks in which the attacker gains numerical advantage [38]. If any inconsistencies are detected, lowering the priority of a given vehicle is used instead of revoking the certificates [39].

Huibin Xu in [40] proposed a way to pluck incorrect information in VANET networks. It consists of examining the consistency of messages transmitted with the actions of the sender. Every vehicle sends its speed and position, and based on the real date, the behavior of that driver is computed [41]. Then, the system estimates whether the vehicle is sending the real information that matches the actual situation on the road. The speed information is verified against the vehicle’s position. The simulations show that this approach effectively detects false information, but requires speedy processing to avoid time-barring events [42].

The method proposed by Yong Hao in [43] bases on rationalizing the location of vehicles based on readings from their GPS sensors. Since two vehicles cannot physically “overlap”, the occurrence of such a situation triggers security mechanisms against attacks. The method does not require the use of additional hardware, which simplifies the car equipment infrastructure [44].

Liang et al. [45] created his solution depending on the similarity of the motion trajectories of Sybil nodes, which are impractical and inappropriate in the real world, believing that Sybil nodes always have the same position and motion trajectories. The solution makes it possible to detect a Sybil attack separately for each car. In order to provide tamper-free periodic digital signatures to neighboring vehicle nodes, it requires the approved infrastructure.

Benkirane [46] suggested a strategy based on a distributed partnership between the roadside units that would allow for a local database providing instant information on vehicles on the road. This knowledge includes the positions in which they associate and the real positions determined based on trilateration between three RSUs using the obtained signal intensity (RSS).

In another early analysis, Xiao et al. [47] proposed a lightweight protection regime focused on estimating the node’s location by analyzing its distribution of signal power. Three functions are delegated to vehicles in this strategy. The claimer is a vehicle that emits beacon signals periodically, including its name and location. The witness is a vehicle within the signal spectrum of the applicant. Witness nodes calculate the claimant’s signal strength and then store this information in their memory along with the claimant’s respective personal data. Thus, they hold a neighbor list that will be transmitted in the next beacon post [48]. When a beacon alert is sent, the vehicles are waiting for a period of time to obtain the previous calculations from the applicant’s witness nodes. Then, they will determine the approximate location of the claimant. RSUs are used to certify the cars’ location behind them to be confident of the car’s path.

Zhou et al. [49] suggested a privacy-preserving detection system without requiring cars to reveal their information on the infrastructure. The Department of Motor Vehicles (DMV) assigns a separate set of pseudonyms instead of assigning precisely one ID to each car, and pseudonyms in the same pool are hashed to a standard value. As a result, a car can use any alias in its pool and maintain its privacy.

Related in forms of privacy security, Sedjelmaci et al. [50] have developed a protocol that helps vehicles detect a Sybil attack in a collaborative manner. The protocol uses party signatures to preserve anonymity and mobility trace similarities. It involves three phases: probing, verifying, and quarantine of nodes.

Mahmood et al. [34] created the solution depending on the similarity of motion trajectories of Sybil nodes, which are impractical and inappropriate in the real world, believing that Sybil nodes always have the same position and motion trajectories. The solution makes it possible to detect a Sybil attack separately for each car. In order to provide tamper-free periodic digital signatures to neighboring vehicle nodes, it requires approved infrastructure.

Finally, one of the potential solutions for identifying the Sybil node may be the resource test. The machine sends complex mathematical problems randomly to the nodes that are supposed to be answered within a given time. Attackers that simulate several nodes are not willing and able to overcome all sent formulas, compromising their simulated cars [19].

The comparison of mentioned methods is presented in Table 2.

## 4. Bogus & Sybil Trust Level & Timestamp (B&STL&T) Algorithm versus Bogus & Sybil Enhanced Behavior Processing & Footprint (B&SEBP&F)

The Bogus & Sybil Trust Level & Timestamp (B&STL&T) algorithm covered in [6] had two different objectives. The first one relies on the car Trust Level (TL), which can be decreased and increased based on the events confirmed/declined by the rest of the vehicles. The stationary network point is responsible for modifying TL. The Sybil detection algorithm is based on the timestamp. As the node reaches another crossroads, its timestamp is renewed after verifying if it was possible for the vehicle to travel the distance at the specified speed.

Based on the experience with the previously implemented Bogus & Sybil Trust Level & Timestamp (B&SEBP&F) algorithm, multiple modifications were applied: an improved system for monitoring drivers behavior in case of an accident, heavy traffic, and police controls. Moreover, the footprint algorithm was implemented in order to prevent Sybil attacks.

### 4.1. B&SEBP&F Algorithm—Solution against Bogus Attack—Monitoring Node Behavior and Interaction with Other Nodes

When a vehicle is in its range, it checks if the trust level needs to be decreased or increased. Car is considered as untrusted when its trust level (TL) drops below 0.5. The second approach, the Bogus & Sybil Enhanced Behavior Processing & Footprint (B&SEBP&F) algorithm, improved the previous one, the Bogus & Sybil Trust Level & Timestamp (B&STL&T), and more events were added to the system.

The first method listed in [6] was the antibogus mechanism that relies on the car’s trust level (TL) so the car confidence. The confidence level for each vehicle varies from 0.0 up to 1.0. This level increases (by 0.1) only if the incident reported by the car is confirmed by another three. This level could be lowered by 0.4 if not enough cars confirm that event within 30 s. The stationary network points maintain the whole grading system. Cars are deemed untrustworthy and removed from the network when their TL falls below 0.5. The current algorithm was expanded and more events were applied to the method in the second approach.

In order to protect from massive bogus attacks, the following events were looked at in greater detail:

- accident,
- heavy traffic,
- speed control.

**Figure 4:**
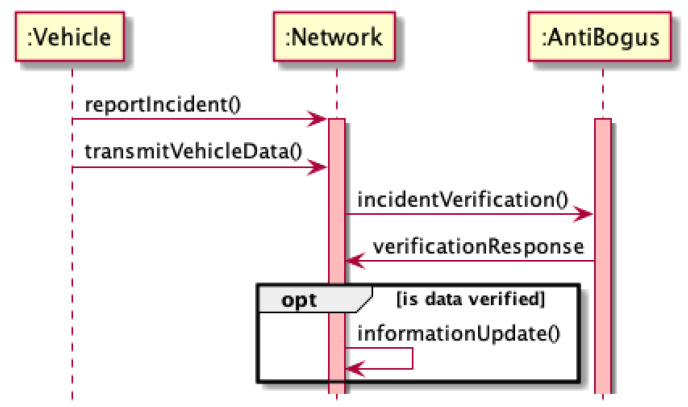
Determination of truthfulness of reported road incident during a bogus attack.

Each of these events is characterized by different vehicle behavior on the road and different expected information from the vehicle. Data provided by the vehicle (i.e., *X* and *Y* coordinates, speed, confidence level, and direction) are analyzed each time to determine the truthfulness of the reported road incident (Algorithm 1 and Figure 4). Each incident or event is sent by the car to the network where is further verified by the antibogus module. Then, if the message was verified correctly, it could appear for the all nodes in the network.
**Algorithm 1:** Determination of truthfulness of reported road incident during a bogus attack.
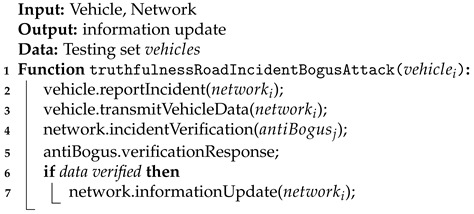


#### 4.1.1. Accident

**Figure 5:**
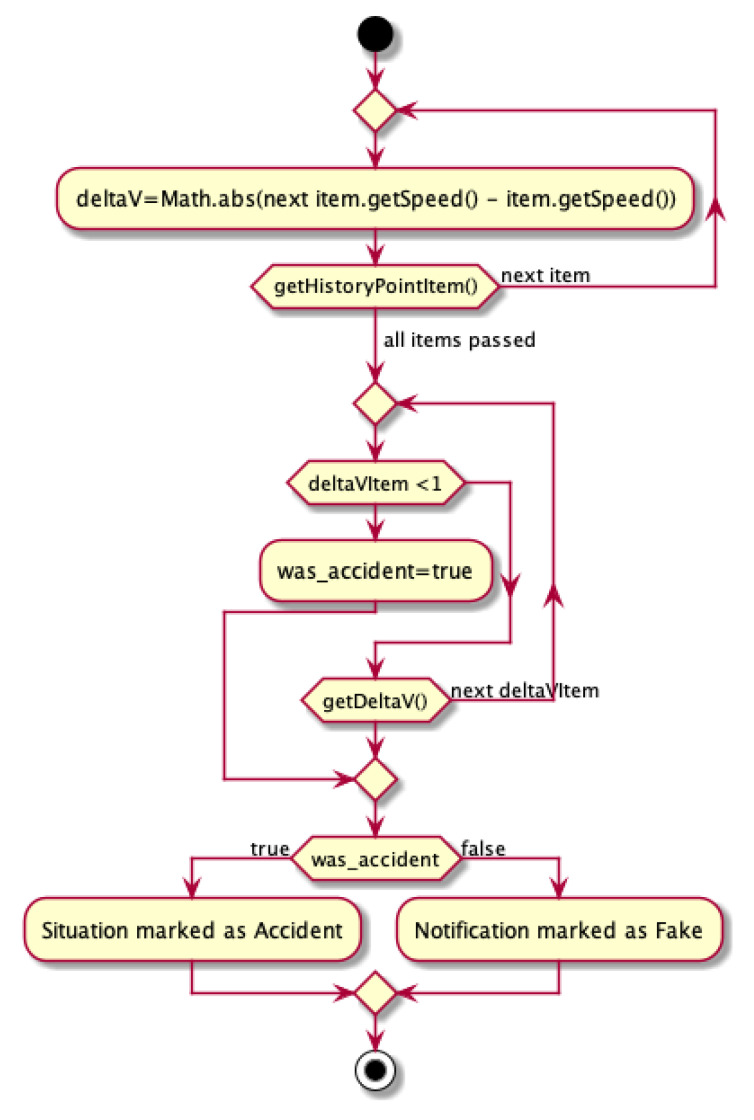
Activity diagram for handling accident detection.

To determine the truth of the reported accident, the vehicle is tested for speed. It is checked whether it suddenly accelerated or braked sharply. If no speed fluctuations have been recorded before, the event is considered as real (Algorithm 2 and Figure 5). Each accident should be reported by all vehicles nearby. Therefore, the system collects all the data and calculates the velocity delta between a particular amount of time for each vehicle. In case that the given delta exceeds the given genuine period, the particular node is excluded from the network and its messages are neglected by any other vehicle, in particular, VANET network.

The first step gathers data about logged vehicle (*deltaV*). Then, that information is compared and it is verified whether the big speed change had happened for each delta log (*deltaVItem*). If the vehicle speed decreased dramatically (the difference between two consecutive speed logs is lower than 1), the event is marked as an accident. Any other incident reported by another vehicle which was not proved in by the rest of vehicles (the speed fluctuations behaves fluently) is marked as fake:

(1) $$[isAccident=\left|nextItem.getSpeed()-item.getSpeed()\right|<1$$

**Algorithm 2:** Handling accident detection during a bogus attack.
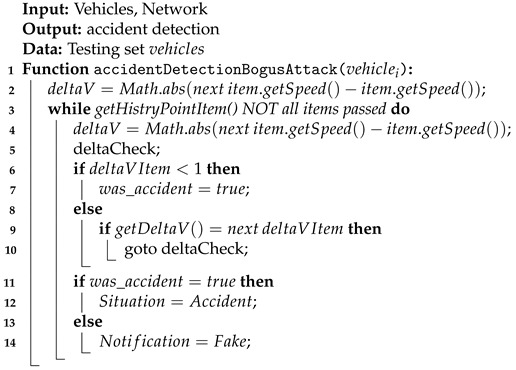


#### 4.1.2. Heavy Traffic

When vehicles are in a traffic jam, they should not be able to reach high speeds. To check if the traffic jam is true, the vehicle speed history before the traffic jam is reported and compared to half of the speed allowed on the road. A report is considered genuine when a sufficient number of vehicle speeds pass the test (Algorithm 3). The system might be resistant to be overridden by attackers, as it simultaneously verifies devices parameters, its behavior, and change of fluency/frequency and compares them with values presented by other nodes.

The system gathers data about the current vehicles’ speeds. In case of a significant difference between the speed limit and a vehicle’s actual speed, the number of traffic jam vehicles increased. Then, the system responds positively to the reported traffic jam if there are enough vehicles to prove it:

(2) $$∥\sum_{i=1}^{20}trafficJamCounter=vehicleSpeedRestriction*0.5>item.getSpeed\left(\right)∥$$

**Algorithm 3:** Handling heavy traffic detection during a bogus attack.
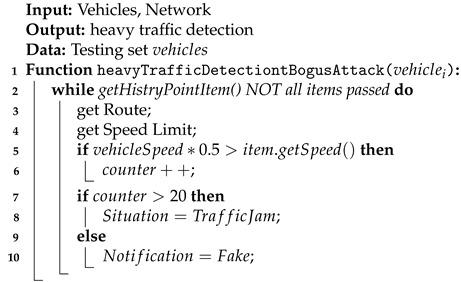



#### 4.1.3. Speed Control

Vehicles’ speeds are tested on a given section to see if they do not drive faster than the permitted speed for a long time. The event is considered real when the appropriate number of speeds that the vehicle had reached prior to reporting the event has passed the test (Algorithm 4).

The system gathers data about current vehicles’ speeds. When cars drive with the lower speed than the particular speed limit, the number of vehicles that might spot police control increases. The system responds positively to the reported police control if there are enough events reported to prove it:

(3) $$∥\sum_{i=1}^{10}roadEventCounter=vehicleSpeedRestriction*0.5>item.getSpeed\left(\right)∥$$

The strength of the proposed solution is that it does not treat all submissions with one measure. Each type of report is checked according to different rules and according to the analysis of the latest statistics of the reporting vehicle. Moreover, this method does not require modification to the vehicles themselves. This solution does not consider the number of submissions, which makes it better for cases of massive attacks. The system allows to monitor a particular node and verify the fluency of its behavior.
**Algorithm 4:** Handling police control detection during a bogus attack.
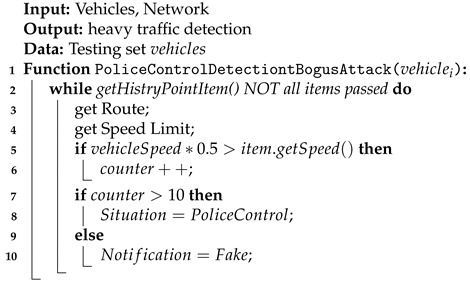



### 4.2. B&SEBP&F Algorithm—Solution against Sybil Attack—Footprint

The solution proposed in [6] is based on the timestamp and nodes credentials. A node (vehicle) drives through a crossroads every time with a given timestamp consisting of a current day and hour. As the node reaches another crossroads, its timestamp is renewed after verifying if it was feasible for the car to travel the given path at the specified speed.

Each roadside unit (RSU) is responsible for sending vehicles within the range of the so-called tag link that contains information about RSU, vehicle position, and timestamp. It should be mentioned that the new timestamp must represent a certain period of time, selected taking into account the location of the RSU (e.g., 500 ms). To protect the network against the possibility of counterfeiting tags, it should be possible to verify its authenticity, e.g., by attaching a digital signature. In this way, the vehicle has a tag link string that describes the history of its movements.

**Figure 6:**
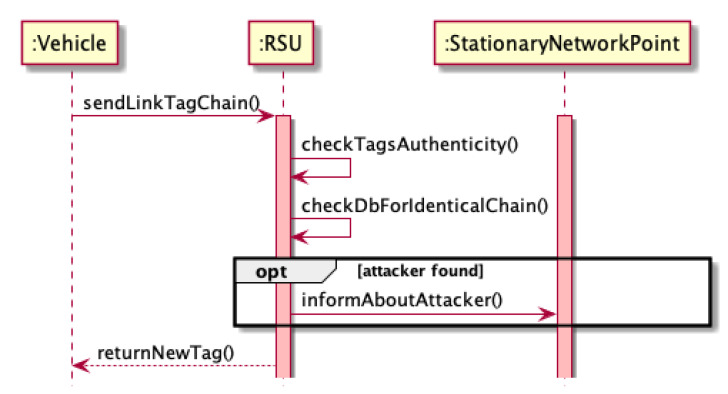
Footprint sequence diagram.

**Figure 7:**
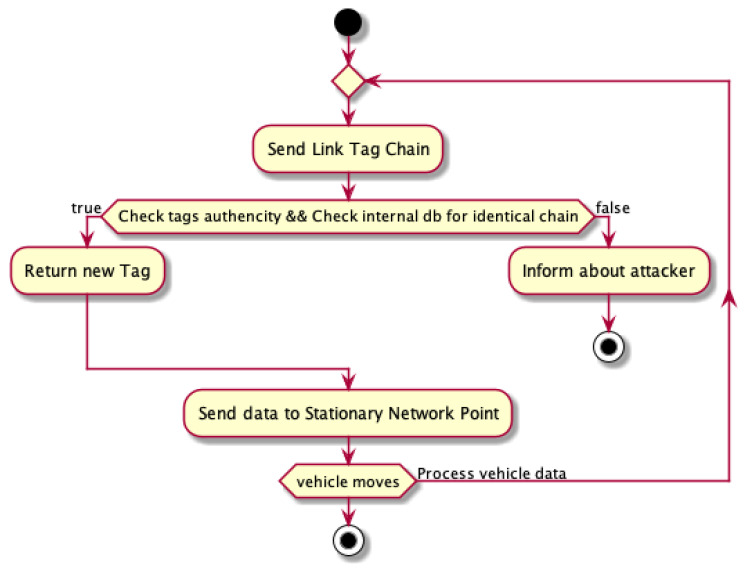
Footprint activity diagram.

Thanks to the use of a link tag system, the network can easily detect the vehicles whose presence is fake. Since this attack is carried out by a physical device (vehicle) transmitting signals on behalf of other nonexistent vehicles, each fake device will also have a tag link chain to be able to confirm its identity for network communication purposes. Since having identical chains describing the vehicle’s trajectory by two real vehicles is relatively small, it can be assumed that having chains with identical tags means that they are bogus vehicles, even if each device reveals its presence with a random delay. The course of communication between the vehicle and the RSU in order to certify its authenticity and receive the next tag could be seen as in the diagrams in Algorithm 5 and in Figure 6 and Figure 7.
**Algorithm 5:** Communication between the vehicle and the RSU in order to certify its authenticity and receive the next tag—footprint during Sybil attack.
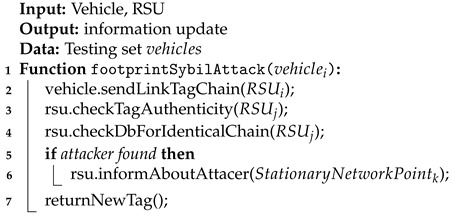



**Figure 8:**
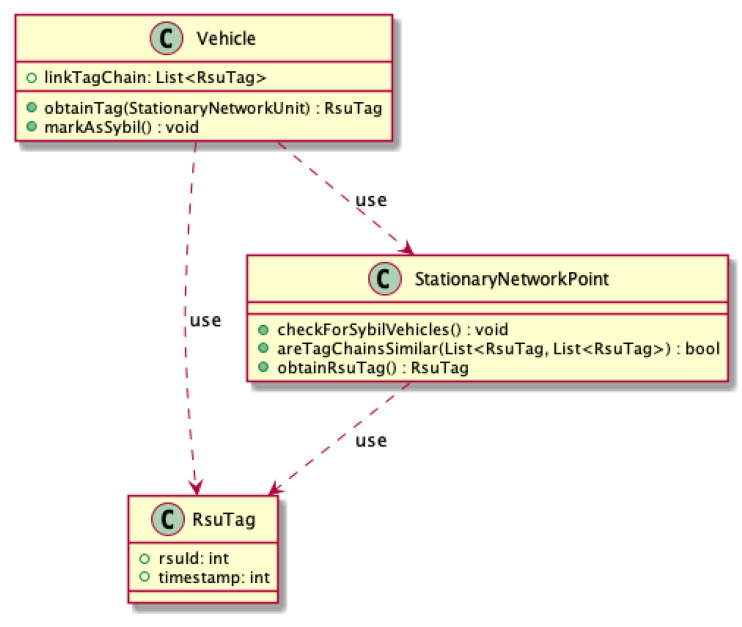
Footprint class diagram.

The *StationaryNetworkPoint* class (Figure 8) is responsible for generating tags for checking in passing vehicles. It also verifies whether the vehicles within the range have different chains. If they are identical, it marks all these vehicles as attackers—it is done by setting a flag on the program’s vehicle object. In fact, such information should be propagated through the entire network. Events reported by vehicles marked as attackers are ignored.

The *Vehicle* class has been equipped with methods allowing for interaction with the implemented tag system. The *SybilVehicle* class, extending it, allows user to simulate an attacker pretending to be any number of vehicles. Internally, such an object has a list of vehicles that it pretends to register their presence in the system, thanks to which they can communicate with the environment (Figure 8).

## 5. Results and Analysis for Sybil/Bogus Prevention Algorithms

**Figure 9:**
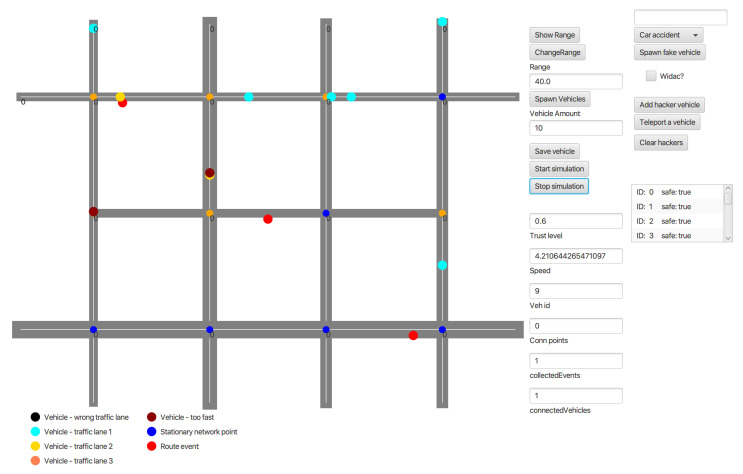
Main view of the application of VANET network to mock the road environment.

The implemented methods for Sybil and bogus attack detection were investigated and checked whether they improve the time of detection of fake cars. In order to test the mentioned algorithms, the VANET network was implemented that mocks the road environment (Figure 9).

The center place in application presents mocked set of crossings and streets. The legend below helps the user with maintaining all events, nodes, and attackers. The right side of the application is settings panel when the user can manipulate with the traffic by spawning new vehicles, fake events, etc.

In order to analyse the artificial VANET network and identify the attacks, the series of experiments were conducted. Each experiment initiated different situations on the roads (car accidents located in random places) and a different ratio of victim vehicles versus attackers nodes.

The application developed previously and described in [6] was improved with the use of new algorithms described in the previous sections. The application can work in offline mode. In order to launch it, JDK 11 is needed. Both simulation and GUI components run on different threads. However, the simulation part is secured from thread synchronization errors.

Furthermore, the system meets the following requirements [6]:

1. Simulation and visualization of car traffic in the street grid. Supporting one-way and both-way streets.
2. Adding any number of cars to the street grid.
3. Starting/stopping simulation.
4. Car data display and modification.
5. Defining static network access points.
6. Interacting individual cars between them and between. Static access points through creating small information exchange networks.
7. Propagating events through VANET network.
8. Showing car signal range visualization.

The map in the application is simply the structure that contains an array list of routes, crossings, events, RSUs, vehicles, and potential attacker objects. Route object contains start point, endpoint, speed limit, and number of lanes in both directions. The initial simulation spawn is defined by the user regular vehicles and several events located in the road’s random places. The system tests the behaviors of the nodes. Therefore, the additional button was implemented to spawn fake vehicles inside the network without interfering with the authorization module. Initially, all vehicles in the network are marked as regular ones. Every vehicle (node) in the network is able to spawn new event to the system; it also shares his information with other vehicles that are close enough (are within specified radius). The application supports spawning fraud messages inside the network, events, and behaves in an abnormal way to disrupt information shared among other vehicles. Those messages are caught and analyzed in the experiments mentioned in the further subsections.

### 5.1. Bogus & Sybil Trust Level & Timestamp (B&STL&T) Algorithm

Table 3 shows the results obtained via the first algorithms that focus on trust level and timestamp only mentioned in [6]. The first column refers to the experiment number. Then, the next two ones refer to the number of user and number of attackers inside the network. Finally, the fourth column shows the ratio between ordinary drivers on the road and attackers.

Table 4 presents the ratio between attackers and attackers and the time needed to identify the first attacker. In the experiments, the ratio between the attackers and the victim nodes vary from 9% up to 100% accordingly. The maximum number of users reached 200 vehicles on the road. The average time needed to identify the first attacker was always 29 ms, and the standard deviation was ±15%.

Table 5 shows how much time the system needs to identify all attackers. Finally, Table 6 shows false positives.

The first series of conducted experiments ratio between victim nodes and attackers varied from 10% up to 100%. The maximum number of users reached 200 vehicles on the road. The average time needed to identify the first attacker was 29 ms, and the standard deviation was ±15%. The time needed to identify all attackers increase proportionally with the increasing attackers’ ratio against victim nodes. The network with the increasing number of computations gathers more data, which simplifies the further computations. Therefore, increasing the number of vehicles up to 175 (75 attackers) leads to a detection time around 116 ms. In the scenario with having 15 cars in the network (and 5 attackers), computation was ended (removing all attackers from the network) within 78 ms. Increasing the number of vehicles by a factor of 11 increased the computation time by a factor of 2.2. The number of false positives (incorrectly marked vehicles as attacker nodes) was always 0 or in some extreme cases 1.

### 5.2. Bogus & Sybil Enhanced Behavior Processing & Footprint (B&SEBP&F) Algorithm

Table 7 presents the results obtained during the next phase after algorithms modification—implementing the Bogus & Sybil Enhanced Behavior Processing & Footprint (B&SEBP&F) algorithm. In Table 7, the multiple experiments with several false positives, detection time of the first attacker, and detection time of all attackers are presented. Moreover, the number of vehicles, number of attackers, and ratio between them was presented as well.

**Figure 10:**
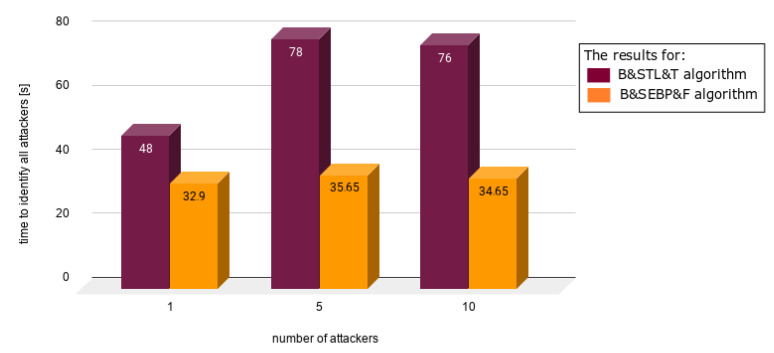
Results from experiments no. 1, 2, and 3 (experiments can be found in Table 3, Table 4, Table 5, Table 6 and Table 7).

**Figure 11:**
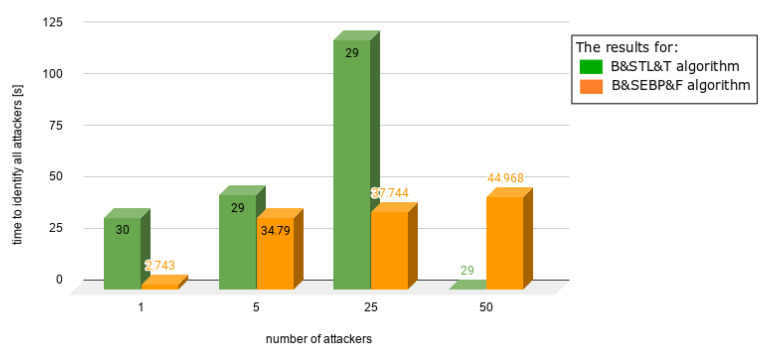
Results from experiments no. 4, 5, 6, and 7 (experiments can be found in Table 3, Table 4, Table 5, Table 6 and Table 7); the graph presents different scenario when the number of users increased significantly as well as number of attackers; the ratios between the attackers and users were 1.96%, 9.05%, 33%, and 50%.

**Figure 12:**
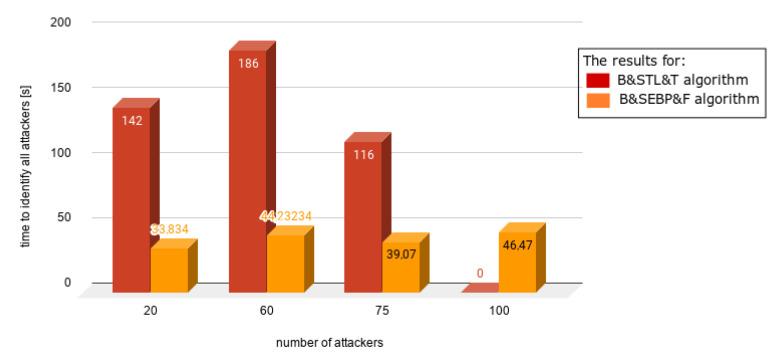
Results from experiments no. 8, 9, 10, and 11 (experiments can be found in Table 3, Table 4, Table 5, Table 6 and Table 7); according to Figure 11 the same number of users were involved—100; the number of attackers varied from 20 up to 100.

**Figure 13:**
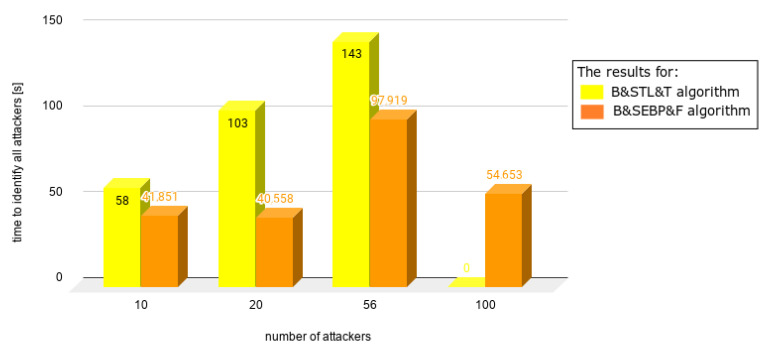
Results from experiments no. 12, 13, 14, and 15 (experiments can be found in Table 3, Table 4, Table 5, Table 6 and Table 7); the graph presents a different scenario in which there are no regular users on the road.

**Figure 14:**
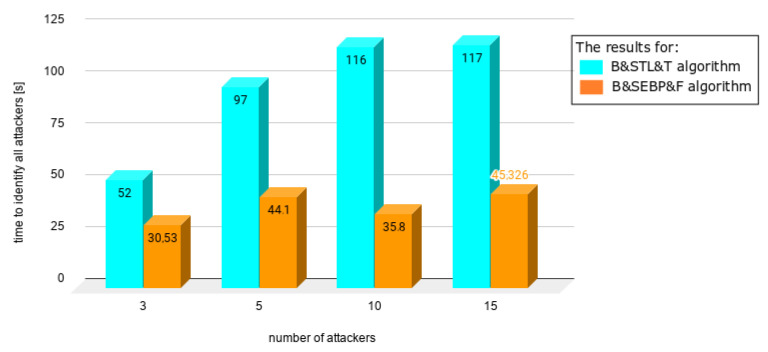
Results from experiments no. 16, 17, 18, and 19 (experiments can be found in Table 3, Table 4, Table 5, Table 6 and Table 7); the graph presents the situation when there are equal numbers of regular users and attackers.

Charts presented in Figure 10, Figure 11, Figure 12, Figure 13 and Figure 14 juxtapose the results from corresponding experiments with and without the algorithm improvements (Bogus & Sybil Enhanced Behavior Processing & Footprint algorithm). Every orange column showcases the results after changes to the application.

The tables presented above show the ratio between number of regular users and attackers and the time needed to identify the first attacker, all attackers for the Bogus & Sybil Trust Level & Timestamp algorithm (Table 3, Table 4, Table 5 and Table 6), and for the Bogus & Sybil Enhanced Behavior Processing & Footprint algorithm (Table 7), respectively.

In Table 3, the different ratios between the attackers and the victim nodes are presented; therefore, the table depicts the experimental description. Table 4 shows the time to identify the first attacker in ms. In these experiments, the ratios between the attackers and the victim nodes vary from 9% up to 100% accordingly. The maximum number of users reached 200 vehicles on the road. The average time needed to identify the first attacker was always 29 ms, and the standard deviation was ±15%. Table 5 depicts the time needed to identify all attackers. Table 6 depicts users incorrectly qualified as attackers (false positives after identifying all attackers).

Figure 10 presents the time needed to identify the first attacker in case of having 1, 5, and 10 attackers, respectively, on the road. The attackers to average users ratio were 9%, 33%, and 50% accordingly. The time needed to identify the first attacker decreased by 50% in comparison with the previously mentioned approach (Bogus & Sybil Trust Level & Timestamp algorithm) [6].

Figure 11 shows different scenarios when the number of users increased significantly as well as the number of attackers. The ratios between the attackers and users were 1.96%, 9.05%, 33%, and 50%. In the case of Figure 12, the same number of users was involved—100. The number of attackers varied from 20 up to 100. The time needed to identify the first attacker decreased significantly up to 78%.

In Figure 13, a different scenario, one in which there are no regular users on the road, is presented. Even without having victim nodes, the time needed to identify the first attacker decreased, due to the implemented method, which focuses on the node behavior. Finally, Figure 14 shows the situation when there is up the equal number of regular users and attackers. The time needed to identify the first attacker was satisfactory.

**Figure 15:**
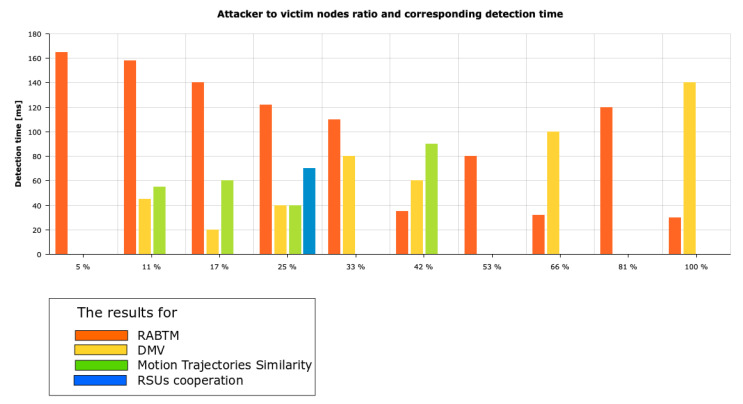
Results from experiments created by the authors of the mentioned works: [19,32,34,37,40,41,43,45,46].

Part of the results obtained from [19,32,34,37,40,41,43,45,46] were coupled into a single table and compared with the algorithm implemented by the authors (Table 8 and Figure 15). The results obtained by other authors varied from 30 up to 140 ms in case of high network load. However, some of the authors obtained significantly worse results, even with the small attacker–victim ratio. The average ratio between normal drivers and attackers varied from 5% up to 100%; the average ratio oscillated about 30%.

In the case of the RABTM method, the ratio varies between 5% and 33%. In comparison to the method presented in the fourth chapter, the number of false positives was the same. However, the results obtained via the B&SEBP&F algorithm was three times better than the mentioned algorithm. In the case of the DMV method, the results obtained are similar to the one acquired by the created algorithm. The motion trajectories similarity algorithm mentioned the test cases when number of attackers reached number of victim nodes. In those scenarios, the B&SEBP&F algorithm gave significantly better results, having a detection time that was three times better. However, the number of false positives was larger than that in methods mentioned by other authors. RSUs cooperation reached results two times worse than those of the presented approach. For 90% of presented experiments, the number of false positives was nearly 0 or detected as about 1 false positive.

After several tests were conducted, the following assumptions were deduced:

- In cases where the number of ordinary vehicles was lower than the number of attackers, the time of detection of the first attacker decreased significantly.
- The time of detection of the first attacker lengthened only in the cases of experiments 12–14, where the number of attackers was higher than the number of ordinary vehicles.
- The time of detecting all attackers was reduced by approximately one-third.
- The speed of cars affects the outcome of experiments.
- The methods described in the literature—specifically, in [19,32,34,37,40,41,43,45,46], the results of which were mentioned in Table 8—represent values worse than the results obtained by the proposed Bogus & Sybil Enhanced Behavior Processing & Footprint (B&SEBP&F) algorithm. However, it is observed that the number of false positives is lower than in the proposed algorithm.

## 6. Discussion and Conclusions

The artificial VANET network (mentioned in Section 4) allows its users to share their information on onroad events and traffic. While this knowledge sharing mechanism (transferring messages between OBUs (onboard units) located inside vehicles) is constructive, it may also be the target of security attacks. There are different ways of misinforming people and different motives for it. As any engineer should be prepared for his device to be targeted, each user should be conscious of the risks of any technology. Via a VANET network or just peer-to-peer networks, several ways to spread disinformation have been determined and some of those mechanisms have been prepared against two forms of attack: Sybil and bogus.

After conducting a series of experiments, a certain phenomenon was noticed in the case of situations in which the ratio of attackers to all vehicles in the network was greater than or equal to 50%; then, the network was able to classify all attackers inside the network correctly. Moreover, after code improvements and analyzing the results from Table 6—column “Time needed to identify all attackers (ms)” with results from Table 7—column “Time to all detection”, there is a significant change in the time of detecting all attackers; this operation is being performed much faster.

Moreover, after implementing changes to the application of artificial VANET network (mentioned in Section 4), previously described the phenomenon of a single attacker not being properly marked disappeared. Cause of this event might be interference of the code changes to the speed of cars. Previously, every car in the network traveled with static speed. Currently, the speed of every car adapts to events on the road.

In cases where the number of ordinary vehicles was lower than the number of attackers, the detection of the first attacker decreased significantly. The time of detection of the first attacker lengthened only in experiments 12–14, where the number of attackers was higher than the number of ordinary vehicles. The time of detecting all attackers was reduced by approximately one-third.

However, after analyzing and comparing the results from Table 6 with results from Table 7, it could be seen that after implementing changes to the algorithm, the number of false positives per run increased. Therefore, there is a potential place that should be improved in future work.

The future of the ITS (intelligent transportation system), where safety and the attack-free environment is required to achieve the desired traffic quality, is the vehicular ad hoc network. However, it is exposed to different attacks due to the open nature of the VANET. The primary objective of this type of network is not only to provide road safety and operation but also to preserve the safety of all network nodes. Securing the VANET is thus a significant challenge. Privacy and authentication seem to be major obstacles with this type of networks. Any assault could represent a significant threat to our life.

## Figures and Tables

**Table 1:** Comparison of current bogus prevention methods.

Author	Method	Advantages	Limitations
Marmol et al. [17]	TRIP	Probabilistic reasoning identifies a node based on the multiple factors.	Difficult to preserve the node’s trust and behavior, as the system does not know that the node is truthful or malicious.
Mahmood et al. [34]	RABTM	Dempster–Shafer evidence theory is used for numerical trust computation.	Probability can model all forms of uncertainty and ignorance.
Kim et al. [14]	CoE	Source priorities could be adjusted by a particular event program in order to mitigate computation.	Require data taken from the multiple sensors in order to provide reliable results.
Ghosh et al. [28]	Post-crash warning	Effectively reduces the false positives and false negatives while effectively detecting misbehavior.	Reserved only for the specific type of event.
Lee et al. [31]	MBRMS	Uses outlier detection technique and misbehaving risk value of the bad node to measure the risk level.	An event observer within one hop of reporter can observe the behavior of reporter but cannot detect behavior of reporter.

**Table 2:** Comparison of current Sybil prevention methods.

Author	Method	Advantages	Limitations
Ruj et al. [37]	MDS	Resistant to massive attacks in which the attacker gains numerical advantages.	Privacy and security problems.
Xu et al. [40]	Pluck Incorrect Information	Effective in detecting false information.	Requires fast processing to avoid time-barring events.
Sedjelmaci et al. [50]	GPS analysis	Does not require the use of additional hardware.	GPS inaccuracy might violate the results.
Liang et al. [45]	Motion Trajectories Similarity	Allows to detect each Sybil node separately.	Requires the approved infrastructure.
Benkirane et al. [46]	RSUs cooperation	Basis of a trilateration provides reliable results.	Require at least three RSUs at a particular crossing.
Xu et al. [47]	Lightweight Protection Regime	Hard to omit the rules by the attacker.	Can be problematic in case of signal power loss.
Zhou et al. [49]	DMV	Does not require cars to reveal their information on the infrastructure.	It does not encourage a misbehaving node to behaving in a proper way and does not punish malicious nodes.
Hao et al. [43]	Rationalizing Positioning Of Vehicles	In case it is confirmed that data delivered do not provide false positives.	GPS inaccuracy might violate the results.

**Table 3:** Bogus & Sybil Trust Level & Timestamp algorithm—parameters of carried out experiments: the number of users, the number of attackers, and the ratio between victim nodes and attackers.

No Experiment	Number of Users	Number of Attackers	Ratio
1	10	1	9.09%
2	10	5	33.33%
3	10	10	50.00%
4	50	1	1.96%
5	50	5	9.09%
6	50	25	33.33%
7	50	50	50.00%
8	100	20	16.67%
9	100	60	37.50%
10	100	75	42.86%
11	100	100	50.00%
12	0	10	100.00%
13	0	20	100.00%
14	0	56	100.00%
15	0	100	100.00%
16	15	3	16.67%
17	15	5	25.00%
18	15	10	40.00%
19	15	15	50.00%

**Table 4:** Bogus & Sybil Trust Level & Timestamp algorithm—time to identify the first attacker in ms.

No Experiment	Ratio	Time to Identify First Attacker [ms]
1	9.09%	48
2	33.33%	57
3	50.00%	19
4	1.96%	35
5	9.09%	36
6	33.33%	23
7	50.00%	25
8	16.67%	35
9	37.50%	26
10	42.86%	28
11	50.00%	26
12	100.00%	22
13	100.00%	25
14	100.00%	10
15	100.00%	-
16	16.67%	30
17	25.00%	29
18	40.00%	29
19	50.00%	29

**Table 5:** Bogus & Sybil Trust Level & Timestamp algorithm—time needed to identify all attackers in ms.

No Experiment	Ratio	Time Needed to Identify All Attackers [ms]
1	9.09%	48
2	33.33%	78
3	50.00%	76
4	1.96%	35
5	9.09%	46
6	33.33%	121
7	50.00%	-
8	16.67%	142
9	37.50%	186
10	42.86%	116
11	50.00%	180 (without the last two)
12	100.00%	58
13	100.00%	103
14	100.00%	143 (to penultimate)
15	100.00%	-
16	16.67%	2
17	25.00%	97
18	40.00%	116
19	50.00%	117

**Table 6:** Bogus & Sybil Trust Level & Timestamp algorithm—users incorrectly qualified as attackers (false positives after identifying all attackers).

No Experiment	Ratio	False Positives after Identifying All Attackers
1	9.09%	0
2	33.33%	0
3	50.00%	1
4	1.96%	0
5	9.09%	0
6	33.33%	0
7	50.00%	-
8	16.67%	0
9	37.50%	-
10	42.86%	1
11	50.00%	0
12	100.00%	0
13	100.00%	0
14	100.00%	0
15	100.00%	0
16	16.67%	0
17	25.00%	0
18	40.00%	4
19	50.00%	0

**Table 7:** The results of experiments after code improvements implementing the Bogus & Sybil Enhanced Behavior Processing & Footprint algorithm.

No Exper.	False Positives after Identifying All Attackers	Time to First Detection	Time to All Detection	No of Ordinary Vehicle	No of Attackers	Attacker to Ordinary Ratio	Crossing No 0	Crossing No 1
1	1	18.73	32.90	10	1	0.0901	3	2
2	2	3.062	35.65	10	5	0.33	7	7
3	1	3.24	34.65	10	10	0.5	11	11
4	1	2.74	2.743	50	1	0.019	9	8
5	1	2.835	34.79	50	5	0.09	9	5
6	2	2.757	37.744	50	25	0.33	36	21
7	5	2.836	44.968	50	50	0.5	52	35
8	9	2.842	33.834	100	20	0.16	36	22
9	11	2.873	44.23234	100	60	0.375	64	46
10	7	2.823	39.07	100	75	0.42	59	36
11	5	2.91	46.47	100	100	0.5	88	58
12	0	28.3	41.851	0	10	1.0	10	6
13	0	28.668	40.558	0	20	1.0	15	11
14	0	26.884	97.919	0	56	1.0	55	53
15	0	24.612	54.653	0	100	1.0	100	83
16	3	2.951	30.53	15	3	0.16	7	7
17	3	2.96	44.10	15	5	0.25	8	8
18	3	2.96	35.80	15	10	0.4	13	12
19	4	5.263	45.326	15	15	0.5	17	17

**Table 8:** Results obtained by the authors of [19,32,34,37,40,41,43,45,46]; the research works examined papers in order to conduct the same experiments that were tested and presented in Table 7.

No Experiment	False Pos. after Ident. All Attack.	Time to All Detection	No. of Ordinary Vehicle	No. of Attackers	Attacker to Ordinary Ratio	Method
1	1	165	95	5	0.05	RABTM
2	2	158	90	10	0.11	RABTM
3	1	140	85	15	0.17	RABTM
4	1	122	80	20	0.25	RABTM
5	1	110	75	25	0.33	RABTM
6	1	45	45	5	0.11	DMV
7	1	40	40	10	0.25	DMV
8	2	35	35	15	0.42	DMV
9	1	32	30	20	0.66	DMV
10	1	30	25	25	1.0	DMV
11	1	20	85	15	0.17	Motion Trajectories Similarity
12	2	40	80	20	0.25	Motion Trajectories Similarity
13	1	60	70	30	0.42	Motion Trajectories Similarity
14	1	80	65	35	0.53	Motion Trajectories Similarity
15	4	100	60	40	0.66	Motion Trajectories Similarity
16	1	120	55	45	0.81	Motion Trajectories Similarity
17	1	140	50	50	1.0	Motion Trajectories Similarity
18	1	55	90	10	0.11	RSUs cooperation
19	1	60	85	15	0.17	RSUs cooperation
20	3	70	80	20	0.25	RSUs cooperation
21	1	80	75	25	0.33	RSUs cooperation
22	1	90	70	30	0.42	RSUs cooperation
